# Synthetic MRI from radiotherapy planning CT images for Head‐and‐Neck Cancer

**DOI:** 10.1002/acm2.70849

**Published:** 2026-10-20

**Authors:** Prabhakar Ramachandran, Akshay C Dhargave, Catherine Jones, Dionee Liefman, Lachlan McDowell, Howard Liu

**Affiliations:** ^1^ Department of Radiation Oncology Princess Alexandra Hospital Woolloongabba Queensland Australia; ^2^ Australian Institute for Bioengineering and Nanotechnology The University of Queensland St Lucia Queensland Australia; ^3^ School of Chemistry and Physics Queensland University of Technology Brisbane Queensland Australia

**Keywords:** deep‐learning, Head‐and‐Neck Cancer, image translation, Pix2PixGAN, radiotherapy planning, Synthetic MRI

## Abstract

**Background:**

Computed Tomography (CT) provides geometric accuracy and electron density information essential for radiotherapy planning; however, it offers limited soft‐tissue contrast. Magnetic Resonance Imaging (MRI) improves tumor visualization but introduces registration uncertainties when acquired separately from the radiotherapy planning CT (pCT). Cross‐modality image synthesis enables the generation of synthetic MRI (sMRI) directly from pCT and may combine the strengths of both modalities, reducing reliance on MRI access.

**Purpose:**

We propose a two‐stage deep‐learning framework for generating sMRI from pCT image‐sets specifically in head‐and‐neck (H&N) cancers, providing soft‐tissue contrast comparable to ground‐truth MR images (gtMRI).

**Methods:**

This study included 60 patient cases with H&N cancer treated with radical radiotherapy between March 2022 and April 2024 for model development (50 for training, 10 for validation), plus 10 additional cases, independent of the development cohort for testing. All patients underwent a standard, non‐contrast pCT scan followed by an MRI acquired on a 3T Siemens MR simulator. Images were deformably‐registered, yielding 10,000 matched CT–MRI slice‐pairs for model training. The proposed model utilizes a two‐stage architecture, a Pix2Pix conditional generative adversarial network (GAN) for coarse sMRI synthesis, followed by a conditional diffusion model for refinement. To test the model's robustness, 10 patient cases with both pCT and contrast‐enhanced CT (ceCT) were used to generate sMRI. Model performance was assessed using structural similarity index (SSIM), multi‐scale structural similarity index (MS‐SSIM), mean absolute error (MAE), and peak signal‐to‐noise ratio (PSNR). In addition, the geometric agreement of the primary (GTVp_70) and nodal (GTVn_70) gross tumor volumes delineated on sMRI vs. gtMRI was independently assessed by two radiation oncologists (ROs) using the Dice similarity coefficient (DSC), 95^th^‐percentile Hausdorff distance (HD95), and average symmetric surface distance (ASSD).

**Results:**

The average MS‐SSIM, SSIM, MAE and PSNR for pCT‐derived sMRI were 0.82 ± 0.02, 0.70 ± 0.03, 0.05 ± 0.01, and 20.42 ± 0.66 dB, respectively. The mean MS‐SSIM and SSIM values for ceCT‐derived sMRI were 0.02 lower than those for the corresponding pCT‐to‐sMRI metrics. For the contour‐based evaluation, sMRI‐derived GTV contours achieved a mean DSC of 0.56 and 0.60 for GTVp_70, and 0.72 and 0.74 for GTVn_70 across the two ROs, relative to gtMRI contours.

**Conclusion:**

Our proof‐of‐concept study demonstrates the feasibility of pCT‐derived sMRI for H&N cancers using a deep‐learning framework. The model produces grayscale patterns and soft‐tissue contrast that closely resemble the gtMRI.

## 1 INTRODUCTION

Head‐and‐neck (H&N) cancers represent a group of tumors originating in the oral cavity, oropharynx, larynx, hypopharynx, nasopharynx, salivary glands and sinonasal region.^1^ Radiotherapy is a standard treatment modality for these cancers. Gross tumor volumes (GTVs) require manual delineation to prescribe high‐dose radiotherapy. Computed tomography (CT) serves as the principal modality for radiotherapy planning, as it provides the essential electron density data required for dose computation.^2^ Nevertheless, CT has limitations in soft‐tissue contrast, and this may impact the accurate definition of tumors and adjacent organs‐at‐risk (OARs).^3^ Magnetic Resonance Imaging (MRI) provides superior soft‐tissue contrast, which assists radiation oncologists (ROs) in defining the extent of both primary and nodal GTVs in H&N cancer.^4^ The absence of electron density information, however, restricts the direct use of MRI datasets for radiotherapy treatment planning, as this information is essential for dose calculation in heterogeneous tissue densities.^5^

Co‐registering MRI to define tumor extent in radiotherapy planning can lead to target volume alterations classified as major in over 50% of cases.^6^ Typically, MRI scans are acquired at different times and under slightly different patient positions; therefore, the co‐registration process can introduce errors related to temporal changes, anatomical shifts and patient motion.^7^, ^8^ Furthermore, MRI is not always readily available due to resource constraints or patient contraindications, and its quality can degrade as a result of variations in acquisition time and motion artifacts.^9^ Cross‐modality image translation is becoming increasingly popular in radiation oncology as it could help streamline clinical workflows and minimize some of the long‐standing problems with imaging, contouring, and treatment planning. In the last few years, deep‐learning techniques have revolutionized imaging in radiation oncology, leading to significant improvements in image quality and the generation of synthetic images for clinical use. For example, they have led to the generation of synthetic CT from MRI, mainly for the brain and pelvis,^10^, ^11^ with reduced artifacts, improved Hounsfield unit (HU) values, reduced noise, and the ability to generate synthetic CT from cone‐beam CT (CBCT) images for adaptive radiotherapy.^12^ Similar progress has been made in translating between Positron Emission Tomography and CT domains, with the goal of reducing reliance on multi‐modality image acquisition and minimizing the registration errors that can arise when images are acquired at different times and under different patient conditions.^13^ The concept of generating synthetic MRI (sMRI) directly from radiotherapy planning CT (pCT) is appealing, as sMRI enables utilizing a single imaging modality (e.g., CT) for treatment planning while preserving soft‐tissue contrast similar to MRI for OAR and GTV definition. This would minimize the need for separate patient appointments, reduce reliance on co‐registration between separately acquired images, and improve the patient experience by avoiding lengthy MRI acquisition times.^14^

Several deep‐learning architectures have been investigated to date for cross‐modality translation. U‐Nets play a key role in image synthesis, thanks to their encoder‐decoder design and skip connections, which capture both global context and precise anatomical details.^15^ Generative adversarial networks (GANs) introduced the concept of using a generator and discriminator to produce realistic images.^16^ Several studies have utilized GANs for cross‐modality synthesis and have shown that they can generate CT‐like images from MRI and CBCT images.^17^, ^18^ GANs mainly rely on paired images for training, which poses a limitation when paired images are not available. This was overcome by the introduction of cycle‐consistent GANs (cycleGANs), which enabled translation between unpaired image domains and have since become a popular approach for MR‐to‐CT synthesis. However, cycleGANs are likely to introduce hallucinated features or geometric distortions as these architectures do not force geometric fidelity.^19^ Of late, diffusion models have emerged as a strong alternative to GANs. These models tend to gradually recreate images by denoising the introduced noise using a trained noise schedule. Studies have demonstrated their ability to generate high‐fidelity medical images.^20^, ^21^ Their application across medical imaging tasks, including cross‐modality synthesis, has been reviewed extensively.^22^, ^23^

In this proof‐of‐concept study, we propose a two‐stage deep‐learning model that utilizes a Pix2Pix‐based conditional GAN (Pix2PixGAN) in Stage‐1 and a diffusion‐based refinement in Stage‐2 to transform pCT image‐sets into sMRI that closely mimics the ground‐truth MRI (gtMRI). This is one of the first studies to explore sMRI in H&N cancers. This technology may assist resource‐limited centers to utilize their pCT images to generate sMRI.

## 2 MATERIALS AND METHODS

### 2.1 Study design and patient cohort

This retrospective study included imaging data from 70 patient‐cases with histologically confirmed H&N cancer treated with curative‐intent radiotherapy, with or without concurrent chemotherapy, between March 2022 and April 2024. Model development used a cohort of 60 patient‐cases, randomly split at the patient level into training (*n =* 50) and validation (*n =* 10) sets using a fixed random seed (42). An additional 10 patient‐cases, independent of the development cohort, were used as the test‐cohort. Ethical approval to conduct this study was received from our institutional Human Research Ethics Committee. Written informed consent for the use of data for research was obtained from all patients included in this study. Table 1 summarizes patient and tumor characteristics of the 70‐patient study‐cohort. The 10 test‐cases were held out entirely from model development and were not used for hyperparameter selection or architectural decisions. Patients with dental implants were also included to ensure the model's suitability across a broad range of routine clinical cases.

**TABLE 1:** Patient and Tumor Characteristics. Values are n (%) unless otherwise stated. GTVp_70 = primary GTV prescribed 70 Gy; GTVn_70 = nodal GTV prescribed 70 Gy; gtMRI = Gadolinium‐enhanced T1‐weighted Dixon MRI sequence; pCT = radiotherapy (standard non‐contrast) planning CT; ceCT = contrast‐enhanced CT.

Characteristic	Training (*n =* 50)	Validation (*n =* 10)	Testing (*n =* 10)
Age (years), median *(range)*	61.5 *(42–87)*	62 *(54–76)*	57.5 *(49–73)*
Sex, *n*: Male / Female	44 / 6	9 / 1	5 / 5
Image sets	gtMRI, pCT	gtMRI, pCT	gtMRI, pCT, ceCT
Dental implants: Yes / No	23 / 27	2 / 8	4 / 6
CT scanner: Philips / Toshiba	29 / 21	7 / 3	6 / 4

Recorded diagnosis/site, *n* (%)			
Base of tongue (C01)	13 (26%)	4 (40%)	4 (40%)
Tonsil (C09.0/C09.8/C09.9)	13 (26%)	3 (30%)	5 (50%)
Other oropharyngeal sites (C10.0/C10.8/C10.9)	12 (24%)	2 (20%)	1 (10%)
Larynx (C32.0/C32.1)	4 (8%)	1 (10%)	–
Other oral cavity/palatal sites (C04.0/C05.1/C06.0/C06.2)	4 (8%)	–	–
Other recorded diagnoses^*^	4 (8%)	–	–

T‐stage (AJCC 8^th^ edition), *n* (%)^†^			
T0	4 (8%)	1 (10%)	–
T1–T2	22 (44%)	2 (20%)	5 (50%)
T3–T4	24 (48%)	7 (70%)	5 (50%)

N‐stage, *n* (%)^†^			
N0–N1	34 (68%)	6 (60%)	3 (30%)
N2–N3	16 (32%)	4 (40%)	7 (70%)

GTV volume, cm^3^			
GTVp_70: Median *(range)*	7.39 *(1.16–116.39)*	10.69 *(2.32–47.98)*	12.65 *(2.30–41.3)*
GTVn_70: Median *(range)*	5.67 *(0.43–135.25)*	28.79 *(2.52–180.76)*	27.50 *(2.50–152.3)*

* Other recorded diagnoses in the training set comprised one overlapping nasopharyngeal lesions (C11.8), two nasal cavity lesion (C30.0), and one combined lower‐third oesophagus/oropharynx diagnosis (C15.5/C10.9).

^†^ T and N categories were grouped from the staging recorded in MOSAIQ. T4A and T4B were included under T3‐T4; N2B, N2C, and N3B were included under N2‐N3.

### 2.2 Imaging acquisition parameters

CT images were acquired on one of two departmental scanners: a Philips^®^ Brilliance^™^ Big Bore CT or a Toshiba^®^ Aquilion^™^ Large Bore. Matched acquisition parameters included: 120 kVp, 2 mm slice thickness, 700 mm extended reconstruction field‐of‐view (FOV), with in‐plane pixel spacing of 1.367 × 1.367 mm and matrix size of 512 × 512. Reconstruction was performed using each vendor's default soft‐tissue convolution kernel (Philips^®^: kernel B; Toshiba^®^: FC13). Each patient underwent a pCT scan followed by MRI within our radiotherapy department using a 3‐Tesla Siemens^®^ MAGNETOM^®^ Vida MR simulator. Axial 3D Gadolinium (Gadovist^®^ 1.0)‐enhanced, T1‑weighted Dixon gtMRI sequences were acquired with 0.9 × 0.9 × 0.9 mm voxels (TE_1_ 1.58 ms, TE_2_ 2.83 ms, TR 4.66 ms, flip angle 10°) and consisted of 200 axial slices for each case. During both CT and MRI acquisitions, patients were immobilized using a treatment mask attached to a flat tabletop placed over the spine array coil. Two UltraFlex Large 18 coils were positioned superiorly, and two Body 18 coils were positioned over the inferior part of the FOV.

The model was trained exclusively on pCT images, reflecting the intended clinical use case. To assess model robustness and generalizability, we also included contrast‐enhanced CT (ceCT) images, acquired at the same imaging session as pCT, in the test‐cohort. This enabled us to assess the model's robustness to variations in image contrast and observe any discernible differences in the generated sMRI.

### 2.3 Image registration and quality assurance

The pCT images were aligned onto gtMRI using Velocity^™^ *v*4.1. Manual rigid alignment was performed to establish a reference, followed by automated rigid (RIR) and deformable registration (DIR) using mutual information‐based similarity metrics. To ensure spatial correspondence between modalities, these deformably‐registered pCT images were resampled in Velocity^™^ to match the gtMRI matrix size (256 × 288) and voxel spacing (0.9375 × 0.9375 mm). Resampled pCT and gtMRI slices were then paired, yielding 10,000 matched CT–MRI slice‐pairs for model development, with resampling ensuring a common spatial resolution across the cohort.

To quantify the accuracy of the CT–MRI DIR used to generate the paired training dataset, a landmark‐based target registration error (TRE) analysis was performed for all cases using 3D Slicer *v*5.2.2.^24^ Four anatomical landmarks clearly identifiable on both CT and MRI were selected per patient: the tip of the odontoid process (C2 dens), the basion (anterior foramen magnum), and the anterior‐inferior vertebral body corners of C3 and C5. These landmarks were chosen as they represent bony structures with high conspicuity on both modalities and span the craniocaudal extent of the H&N planning volume, thereby providing a representative sampling of the registration field. For each patient, corresponding landmark coordinates were placed independently on gtMRI and resampled pCT volumes. The 3D Euclidean TRE was computed for each landmark pair as:

$$\mathrm{TRE}\;=\sqrt{\left(\Delta x^{2}\right)+\left(\Delta y^{2}\right)+\left(\Delta z^{2}\right)}$$

where Δx, Δy, and Δz are the co‐ordinate differences between the resampled pCT and gtMRI landmark positions in the left‐right, anteroposterior, and craniocaudal directions, respectively. A per‐patient mean TRE was derived by averaging across the four landmarks, and summary statistics were computed across all patients.

Prior to training, matched CT–MRI slice‐pairs’ intensities were normalized using percentile‐based clipping (1^st^–99^th^ percentile) and linearly rescaled to [0, 1] per‐image using min‐max normalization. This approach robustly handles outlier intensities arising from dental artifacts and air regions without requiring manual windowing.

### 2.4 Model development: data pre‐processing and training

We used a two‐stage CT‐to‐MRI synthesis framework to train our model, as schematically illustrated in Figure 1. In Stage‐1, Pix2PixGAN,^25^ consisting of a generator and a discriminator, was used to generate coarse sMRI from resampled pCT. The generator housed a 2D encoder‐decoder U‐Net architecture^15^ with skip connections, convolutional down‐sampling, bilinear up‐sampling followed by Conv2D refinement, BatchNorm2D, and ReLU activations. A 70 × 70 PatchGAN discriminator classified local image‐patches as real or synthetic, and provided adversarial feedback to guide generator training. The generator was optimized using a composite objective comprising adversarial loss,^16^ L1 reconstruction loss, VGG perceptual loss,^26^ and gradient consistency loss,^27^ promoting image fidelity, soft‐tissue texture preservation, and sharper anatomical boundaries, respectively. Training used the Adam optimizer^28^ with batch size = 4, learning‐rate = 2 × 10^−4^, *β_1_* = 0.5, and *β_2_* = 0.999. To prevent data leakage,^29^ training and validation sets were partitioned at the patient‐level. The validation subset monitored convergence, with mean square error (MSE), peak signal‐to‐noise ratio (PSNR), and structural similarity index (SSIM) computed after every epoch. Early stopping and learning‐rate reduction on plateau were based on validation MSE. Stage‐1 was trained for a maximum of 200 epochs with a patience of 30. The checkpoint with the lowest validation MSE was selected for subsequent Stage‐2 training.

**FIGURE 1:**
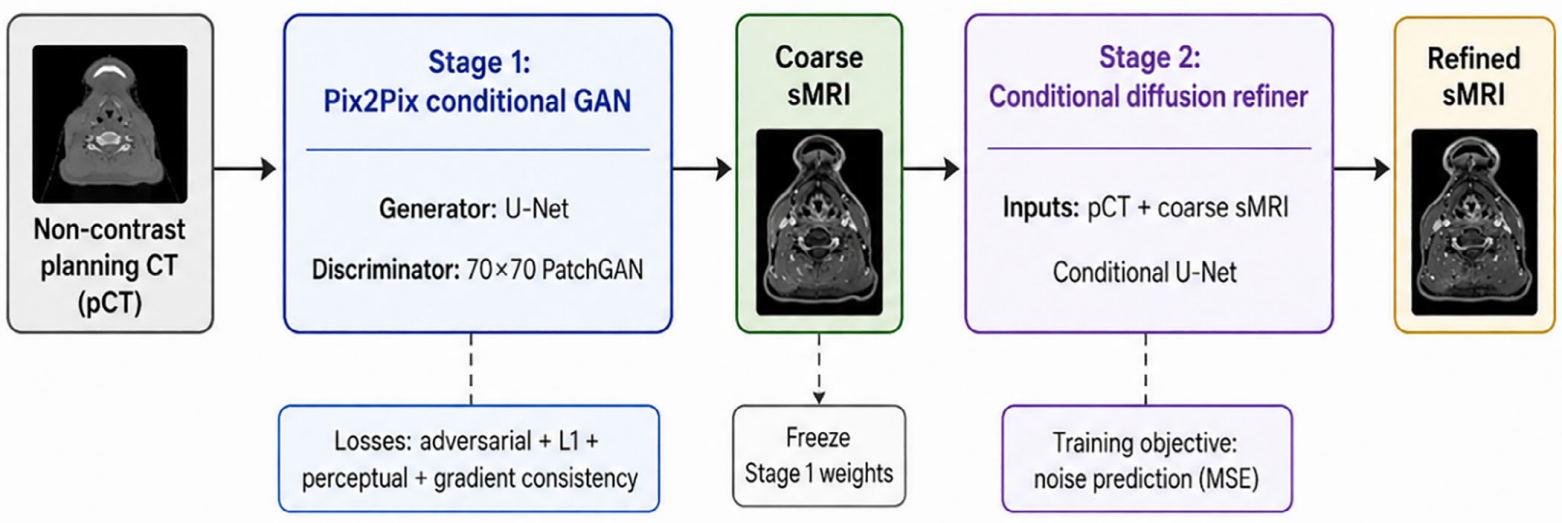
Schematic representation of the two‐stage Pix2PixGAN–diffusion CT‐to‐MRI synthesis framework. A Pix2Pix‐based conditional GAN creates a coarse sMRI from pCT in Stage‐1. After the Stage‐1 generator weights are frozen, a conditional diffusion model is fed the coarse sMRI and the initial pCT for refinement, resulting in the final sMRI.

In Stage‐2, the coarse sMRI estimates generated by the pre‐trained Pix2PixGAN were then fed into a conditional diffusion model. The diffusion refiner housed a U‐Net backbone with residual blocks, GroupNorm,^30^ SiLU activation,^31^ skip connections, and a self‐attention bottleneck.^32^ At every diffusion training time‐step *t*, the U‐Net refiner received a three‐channel input composed by concatenating noisy gtMRI (at noisy time‐step *t*), resampled pCT, and Stage‐1 coarse sMRI. The encoder channel progression was 3 → 64 → 128 → 256 → 512 → 1024, with 256‐dimensional sinusoidal time‐step embeddings projected through a two‐layer multilayer perceptron with Gaussian error linear unit activation. Before diffusion training, the normalized MRI was further rescaled from [0, 1] to [−1, 1], as standard practice for denoising diffusion probabilistic models (DDPMs). Gaussian noise was progressively added over *t* = 1000 training time‐steps using a linear beta schedule ranging from *β_1_* = 1 × 10^−4^ to *β_t_* = 0.02. The model was trained to predict the added Gaussian noise using a mean square error (MSE) denoising objective.^33^ Training was optimized using AdamW,^34^ with batch size = 4, learning‐rate = 1 × 10^−4^, weight decay = 0.01, and gradient norm clipping at 1.0. Stage‐2 was trained for a maximum of 100 epochs with an early‐stopping patience of 15. The checkpoint achieving the lowest validation MSE was retained for subsequent evaluation.

During inference, the reverse diffusion process was initialized by adding partial noise to the Stage‐1 coarse sMRI at *t* = 100 (10% of the full diffusion chain), rather than initializing the process from pure Gaussian noise. The resampled pCT and Stage‐1 coarse sMRI remained fixed as conditioning inputs, while the noisy image estimate was iteratively denoised using 50 ancestral DDPM sampling steps to produce the final sMRI. Initializing refinement from a partially noised Stage‐1 estimate constrained the generative search space, thereby reducing inference time and limiting the potential for anatomically implausible image generation. No data augmentation was applied during either training stage; the 10,000 matched CT−MRI slice‐pairs provided sufficient anatomical diversity across imaging levels, patient positioning variations, and dental artifact presentations. Training was performed on an NVIDIA^®^ RTX^™^ A4500 GPU (16 GB GDDR6), with an inference time for the full two‐stage pipeline of approximately 3–5 minutes per CT volume.

### 2.5 Model evaluation

Prior to metric computation, both gtMRI and sMRI were intensity‐normalized to [0, 1] per image and histogram matching was applied to the sMRI using gtMRI as the reference to remove residual global intensity scale differences. These generated sMRI were then quantitatively evaluated against the gtMRI using standard image‐translation fidelity metrics: SSIM,^35^ multi‐scale SSIM (MS‐SSIM),^36^ mean absolute error (MAE),^19^ and PSNR. All metrics were computed within the patient body region only, with background air outside the scanned volume excluded using CT‐based intensity thresholding, ensuring that reported values reflect performance within clinically relevant anatomy rather than being influenced by uninformative background voxels. Given that MAE and PSNR are computed under per‐image normalization and over different spatial extents, they capture complementary aspects of image fidelity and are best interpreted independently rather than directly compared against each other.

SSIM is widely used in medical image synthesis as it correlates well with human visual perception of structural fidelity and is defined as:

$$\mathrm{SSIM}\;\left(x,y\right)=\frac{\left(2\mu_{x}\mu_{y}+C_{1}\right)\left(2\sigma_{\mathrm{xy}}+C_{2}\right)}{\left(\mu_{x}^{2}+\mu_{y}^{2}+C_{1}\right)\left(\sigma_{x}^{2}+\sigma_{y}^{2}+C_{2}\right)}$$

where:

- x = gtMRI patch (or image),
- y = sMRI patch (or image),
- $\mu_{x},\mu_{y}$ = mean intensities of x and y,
- $\sigma_{x}^{2},\sigma_{y}^{2}$ = variances of x and y,
- $\sigma_{xy}$ = covariance between x and y,
- $C_{1},C_{2}$ = small positive constants to stabilize the division

The above relation compares luminance, contrast and structural information between two images and provides a perceptually meaningful similarity score. A value of 1 indicates both images are identical, and lower values reflect increasing differences in intensity distribution and structural detail.

We also used MS‐SSIM defined by Wang et al.^36^ over four progressively down‐sampled image resolutions, and the resultant values were combined using a weighted geometric mean. This allows SSIM to capture structural fidelity not only at the original resolution but also across coarser spatial scales and provides a more perceptually aligned assessment of image similarity.

$$\mathrm{MS}-\mathrm{SSIM}\;\left(X,Y\right)=\prod_{j=1}^{M}{\left[\mathrm{SSI}M_{j}\left(X_{j},Y_{j}\right)\right]}^{w_{j}}$$

where:

- $X_{j}$ and $Y_{j}$ = the sMRI and gtMRI image‐patches at scale j,
- $\mathrm{SSI}M_{j}$ = SSIM computed at that scale
- $w_{j}$ = the normalized multi‐scale weights (0.0517,0.3295,0.3462,and0.2726), and
- M = the number of scales used (four in this study).

The original formulation of MS‐SSIM^36^ uses five dyadic scales with weights (0.0448,0.2856,0.3001,0.2363,0.1333). In this study, only the first four scales were used, because the 256 × 288 slices reduce to 16 × 18 pixels at the fifth scale, which is too small for a meaningful 7 × 7 SSIM window. The first four weights were therefore retained and renormalized to sum to one, giving the values of w_j_ listed above. Since these normalized weights differ from the original formulation, the absolute MS‐SSIM values reported here are not directly comparable with those obtained from five‐scale implementations, although the relative emphasis across scales is preserved. The use of four scales still gives a broader view of structural agreement than single‐scale SSIM alone.

To quantify the absolute intensity differences between the generated sMRI and the gtMRI, we utilized the standard MAE defined as:

$$\mathrm{MAE}\;=\frac{1}{N}\;\sum_{i\;=1}^{N}\left|x_{i}-y_{i}\right|$$

where:

- $x_{i}$ and $y_{i}$ = the intensities of the sMRI and gtMRI at voxel i respectively,
- N = the total number of voxels within the image.

A lower value indicates that the sMRI intensities match closely to the gtMRI intensities.

To assess the voxel‐wise intensity fidelity between the sMRI and gtMRI, we utilized PSNR as defined below:

$$\mathrm{PSNR}\;=10\cdot \log_{10}\left(\frac{\mathrm{MA}X^{2}}{\mathrm{MSE}}\right)$$

where:

- MAX = maximum possible intensity value
- MSE = mean squared error between the two images.

### 2.6 Ablation study

To quantify the contribution of the diffusion‐based refinement stage, an ablation study was performed in which the same 10 test‐cases were evaluated using Stage‐1 (Pix2PixGAN) output alone, without Stage‐2 processing. All four image‐fidelity metrics (MS‐SSIM, SSIM, MAE, PSNR) were computed for both Stage‐1 and the full two‐stage pipeline across every test‐case, enabling direct quantitative comparison. Statistical significance of the metric differences was assessed using the Wilcoxon signed‐rank test, with *p* < 0.05 considered to be significant.

### 2.7 Contour‐based evaluation

To evaluate the clinical utility of the sMRI, contour‐based analysis was performed on all ten test‐cases using two GTV sub‐volumes: the primary GTV at 70 Gy (GTVp_70) and the nodal GTV at 70 Gy (GTVn_70). The gtMRI contours were retrieved from radiotherapy treatment planning DICOM RTSTRUCT files generated by the treating ROs, with consensus as per our departmental guidelines. Before RO review, the gtMRI and the generated sMRI volumes were rescaled from the normalized [0, 1] intensity range to the [0, 255] display range. No histogram matching was applied to the sMRI volumes shown to the ROs. Two evaluating ROs (RO_1_ and RO_2_) independently contoured all 10 test‐cases on the sMRI. RO_1_ had originally been involved in delineating the ground‐truth volumes for a subset of these cases at the time of treatment, whereas RO_2_ had no prior involvement with any of the ground‐truth contours. During sMRI contouring, both evaluating ROs were blinded to patient identity, corresponding diagnostic or pCT images, and clinical context. It is also worth noting that the sMRI contouring took place over two years after the original ground‐truth contours were delineated, which should limit any recall bias for cases where the same RO was involved in both. Contour agreement was quantified using Dice Similarity Coefficient (DSC), 95^th^‐percentile Hausdorff Distance (HD95), and Average Symmetric Surface Distance (ASSD). Summary statistics (mean ± standard deviation (SD), median, interquartile range (IQR), minimum, and maximum) were computed across all the test‐cases for every GTV structure.

## 3 RESULTS

Landmark‐based TRE analysis performed across all cases using four anatomical landmarks per case (C2 Dens Tip, C3 Vertebra, C5 Vertebra, and Basion) across the cohort revealed that the mean TRE was 2.15 ± 1.19 mm (median 1.95 mm, IQR 1.14–3.00 mm; range 0.33–4.94 mm). The minimum per‐case mean TRE was 1.61 mm, and the maximum was 2.98 mm.

The proposed model generated sMRI volumes with tissue contrast and anatomical detail that closely resembled the gtMRI. Figure 2 illustrates pCT‐derived sMRI with improved soft tissue contrast relative to pCT for a representative test‐case slice, clearly visualizing muscles, fat and submandibular glands. Figure 3 demonstrates that the model preserves anatomical geometry and soft‐tissue boundaries across axial levels from the nasal septum through the supraclavicular fossa.

**FIGURE 2:**
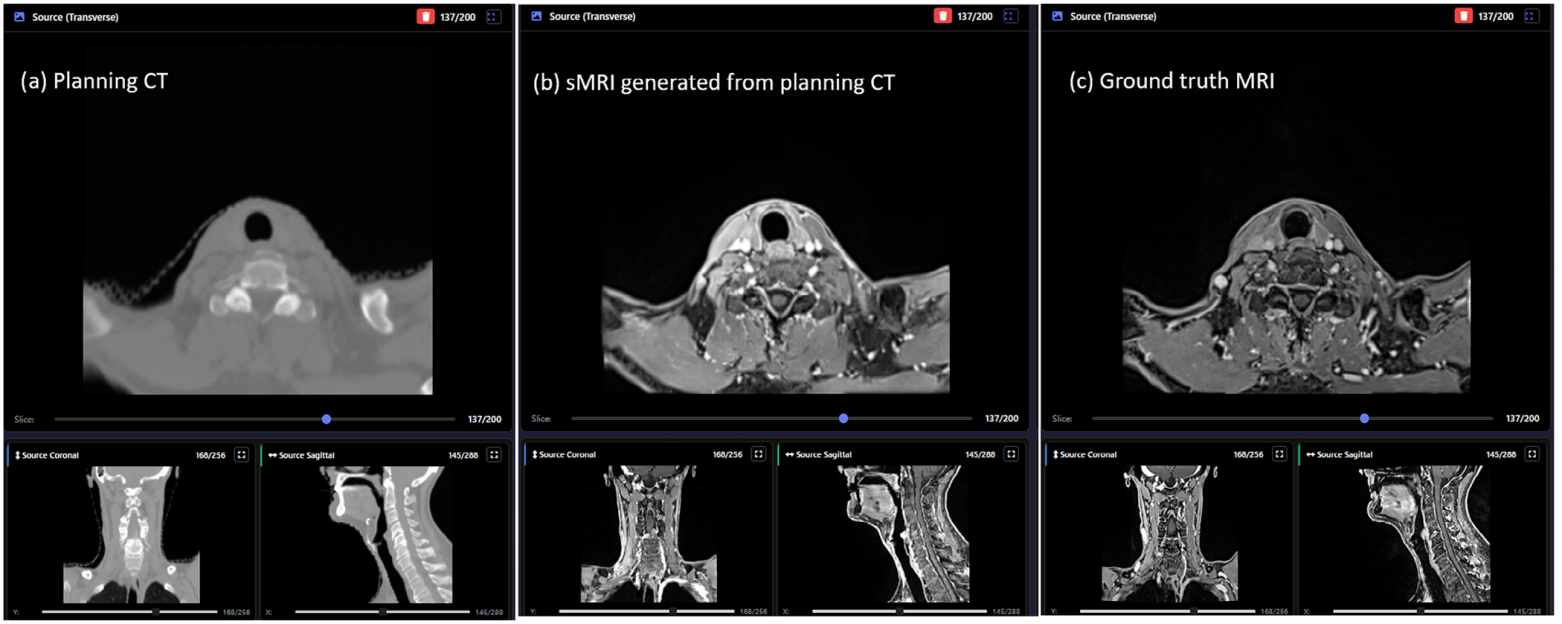
Representative pCT‐derived sMRI generation for a test‐case. (a) Input pCT dataset, deformably‐registered and resampled to the corresponding gtMRI geometry; (b) pCT‐derived sMRI using the full Pix2PixGAN–diffusion pipeline; (c) Corresponding gtMRI. Axial, coronal, and sagittal views are shown at matched anatomical locations/image slices.

**FIGURE 3:**
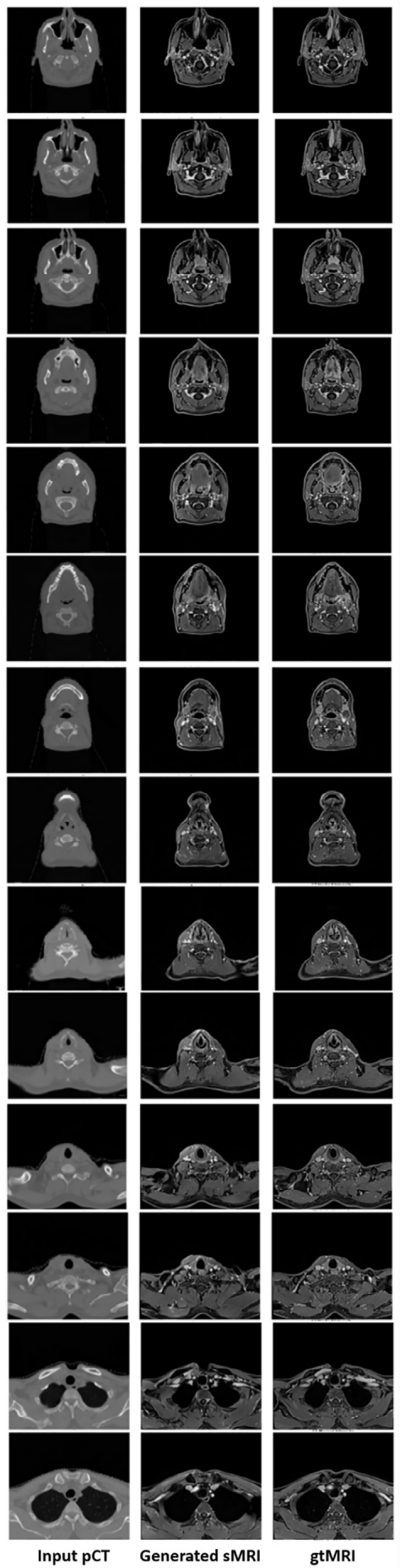
Comparison of gtMRI against pCT‐derived sMRI. Representative axial slices from a single test‐case are shown from superior to inferior anatomical levels. Each row presents the input resampled pCT (left), the generated sMRI (center), and the corresponding gtMRI (right).

Quantitative performance across the 10 test‐cases is shown in Table 2. The model achieved a mean MS‐SSIM of 0.82 ± 0.02 (95% CI [0.81, 0.84]), demonstrating strong structural correspondence between pCT‐derived sMRI and gtMRI. Single‐scale SSIM values were lower with a mean of 0.70 ± 0.03 (95% CI [0.68, 0.72]), primarily due to limited MRI FOV at inferior anatomical levels, typically below supraclavicular slices. Mean MAE was 0.05 ± 0.01 (95% CI [0.04, 0.06]), indicating relatively small voxel‐wise intensity differences between sMRI and gtMRI. The corresponding PSNR averaged 20.42 ± 0.66 dB (95% CI [19.94, 20.89]), indicating stable reconstruction fidelity across the cohort.

**TABLE 2:** Quantitative performance of pCT‐derived sMRI for the test‐cohort, *n =* 10 patients. Slice‐level metrics were averaged within each patient; values are summarized across patients. SSIM = (single‐scale) structural similarity index; MS‐SSIM = multi‐scale structural similarity index; MAE = mean absolute error; PSNR = peak signal‐to‐noise ratio; CI = confidence interval.

Metric	Mean ± SD	Range	95% CI		Median
			Lower	Upper	
**MS–SSIM**	0.82 ± 0.02	0.79–0.85	0.81	0.84	0.82
**SSIM**	0.70 ± 0.03	0.65–0.74	0.68	0.72	0.70
**MAE**	0.05 ± 0.01	0.04–0.06	0.04	0.06	0.04
**PSNR (dB)**	20.42 ± 0.66	18.78–21.23	19.94	20.89	20.59

*Note*. The CI of the mean assumes sample means follow a *t*–distribution with *N* – 1 = 9 degrees‐of‐freedom.

The ablation study comparing Stage‐1 (Pix2PixGAN alone) against the full two‐stage pipeline across matched slices from all 10 patients demonstrated statistically significant improvement attributable to the diffusion‐based refinement stage. Stage‐2 improved MAE by 0.85%, SSIM by 0.73% and PSNR by 0.32%. All improvements were statistically significant (Wilcoxon signed‐rank test, *p* < 0.001 for all metrics), with medium‐to‐large effect sizes (*r* = 0.47 – 0.60). Notably, 70 – 80% of individual slices showed consistent improvement across all metrics, confirming that the refinement effect is systematic rather than incidental. Figure 4 presents the patient‐wise distribution of MS‐SSIM values for sMRI across every test‐case. Each boxplot summarizes the per‐slice MS‐SSIM for an individual patient, with a black diamond indicating the patient's mean. The mean value is close to the overall cohort average of 0.82 (indicated by the dashed line), suggesting that the model performs consistently across individuals. Most test‐cases exhibit MS‐SSIM values between 0.78 and 0.88, with the upper quartiles often exceeding 0.85. This reflects strong agreement between the pCT‐derived sMRI and gtMRI. It also illustrates that none of the patients deviates markedly from the group trend. This suggests that the model generalizes well across varying anatomical presentations.

**FIGURE 4:**
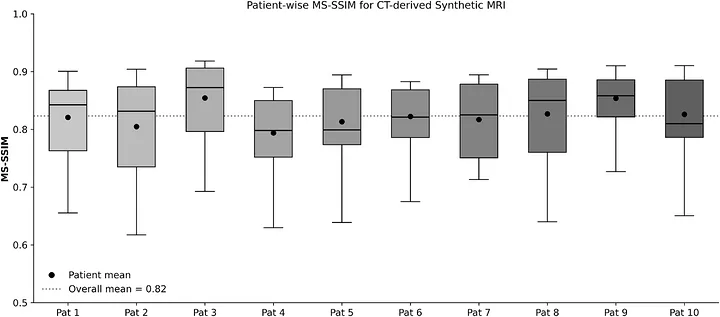
Patient‐wise boxplot of MS‐SSIM distributions for all 10 test‐cases (pCT‐derived sMRI). Boxes represent the patient‐specific interquartile range, solid horizontal lines indicate the median, and whiskers represent the observed range. Black diamond markers indicate the patient‐specific mean, while the dashed horizontal line represents the overall test‐cohort mean.

For every test‐case, pCT, ceCT, and gtMRI scans were acquired on the same day. Both pCT and ceCT were independently used to generate sMRI using the full two‐stage pipeline and evaluated against the same gtMRI reference. Figure 5 illustrates the MS‐SSIM distribution using a violin‐plot between pCT and ceCT datasets. The mean MS‐SSIM, SSIM, MAE and PSNR values for ceCT‐derived sMRI when compared against gtMRI were 0.80 ± 0.08 (median: 0.82; range: 0.55–0.90), 0.68 ± 0.09 (median: 0.70; range: 0.44–0.80), 0.05 ± 0.01 (median: 0.04; range: 0.02–0.10), and 19.90 ± 1.06 dB (median: 20.0; range: 16.60–21.90), respectively. The 0.02 absolute difference in MS‐SSIM between pCT‐derived and ceCT‐derived sMRI volumes is theoretically due to the model being trained exclusively on pCT images. Upon reviewing Figure 6, pCT‐derived sMRI demonstrated slightly higher similarity to the gtMRI than ceCT‐derived sMRI.

**FIGURE 5:**
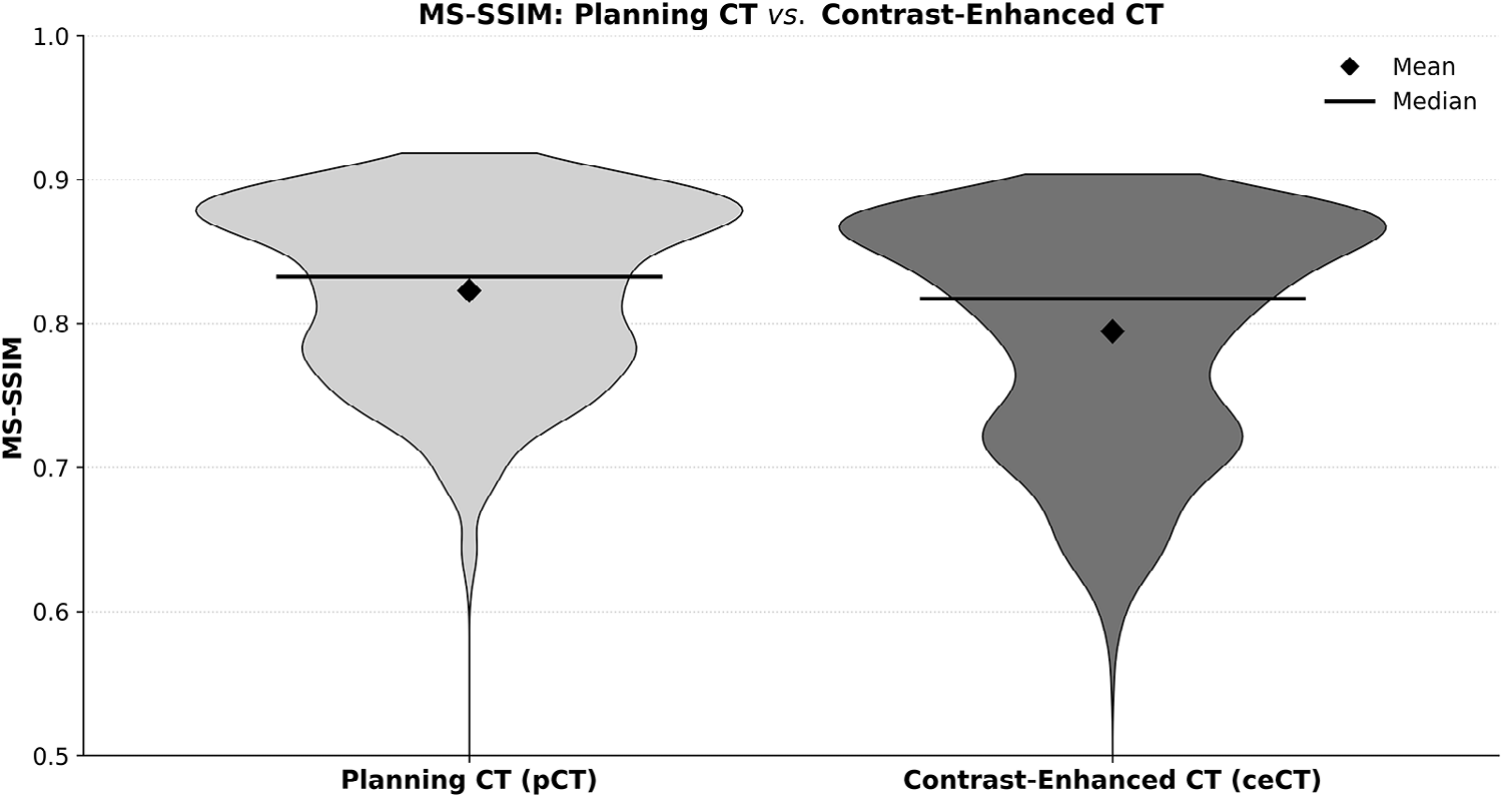
Violin‐plot comparing the MS‐SSIM distributions with pCT‐derived sMRI and ceCT‐derived sMRI. Black diamond markers and solid horizontal lines indicate the mean and median of the entire test‐cohort, respectively.

**FIGURE 6:**
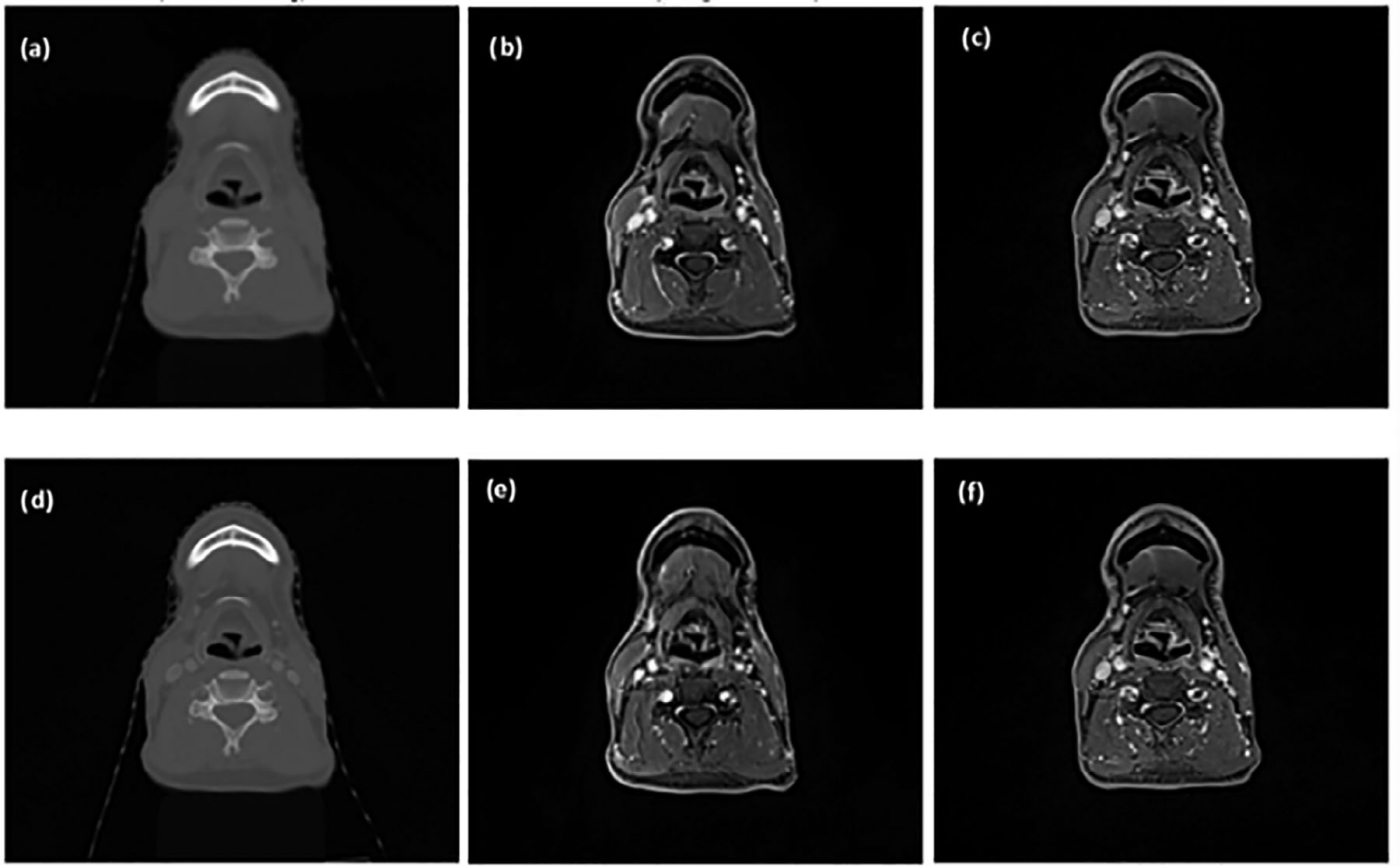
Side‐by‐side comparison of pCT‐derived sMRI and ceCT‐derived sMRI. (a) Input pCT; (b) pCT‐derived sMRI; (c) Corresponding gtMRI; (d) Input ceCT; (e) ceCT‐derived sMRI; (f) Corresponding gtMRI.

An exploratory sub‐group comparison by CT scanner vendor showed a small observed difference of 0.002 in MS‐SSIM between Philips^®^ Brilliance^™^ Big Bore (mean 0.82 ± 0.06) and Toshiba^®^ Aquilion^™^ Large Bore (mean 0.82 ± 0.07) acquisitions of pCT; however, with only 6 and 4 test‐cases per vendor respectively, this comparison is underpowered and should not be interpreted as evidence of multi‐vendor generalizability. All pCT images shared a uniform native in‐plane spacing of 1.367 mm and were resampled to a uniform 0.9375 × 0.9375 mm resolution prior to model input.

Table 3 summarizes the geometric agreement between the GTV contours delineated on sMRI and those on gtMRI for the primary (GTVp_70) and nodal (GTVn_70) target volumes, assessed independently by two evaluating ROs (RO_1_ and RO_2_) across the test‐cases. Agreement was consistently higher for GTVn_70 (overall DSC 0.73 ± 0.08, 95% CI [0.67, 0.79]) than for GTVp_70 (overall DSC 0.58 ± 0.13, 95% CI [0.49, 0.68]), with correspondingly lower HD95 and ASSD values. Both evaluating ROs produced closely comparable metrics for each GTV, indicating that sMRI‐based delineation was robust to inter‐observer variation. Figure 7 presents a representative comparison of GTVp_70 and GTVn_70 contours delineated on pCT‐derived sMRI against those on corresponding gtMRI for an illustrative atest‐case.

**TABLE 3:** Test‐cases’ summary of GTV geometric segmentation accuracy metrics for GTVn_70 and GTVp_70 contours on gtMRI by the treating ROs and those on sMRI independently contoured by evaluating ROs (RO_1_, RO_2_). DSC = Dice similarity coefficient, HD95 = 95^th^‐percentile Hausdorff distance, ASSD = average symmetric surface distance, CI = confidence interval.

Target Volume	Metric	RO	Mean ± SD	Range	95% CI		Median
					Lower	Upper	
**GTVp_70**	DSC	RO_1_	0.56 ± 0.13	0.41–0.71	0.47	0.66	0.55
		RO_2_	0.60 ± 0.13	0.44–0.77	0.51	0.70	0.56
		Overall	**0.58 ± 0.13**	**0.41**–**0.77**	**0.49**	**0.68**	**0.56**
	HD95 (mm)	RO_1_	14.63 ± 7.35	3.84–29.06	9.38	19.89	15.02
		RO_2_	10.98 ± 7.13	2.28–22.52	5.89	16.09	7.64
		Overall	**12.81 ± 7.29**	**2.28**–**29.06**	**7.59**	**18.02**	**12.38**
	ASSD (mm)	RO_1_	4.42 ± 1.87	1.36–8.15	3.09	5.76	4.70
		RO_2_	3.71 ± 1.72	1.11–6.27	2.48	4.95	3.41
		Overall	**4.07 ± 1.79**	**1.11**–**8.15**	**2.79**	**5.35**	**4.50**
**GTVn_70**	DSC	RO_1_	0.74 ± 0.08	0.57–0.83	0.68	0.80	0.74
		RO_2_	0.72 ± 0.09	0.54–0.83	0.66	0.78	0.73
		Overall	**0.73 ± 0.08**	**0.54**–**0.83**	**0.67**	**0.79**	**0.74**
	HD95 (mm)	RO_1_	12.09 ± 7.41	3.14–23.24	6.79	17.39	12.02
		RO_2_	10.41 ± 8.92	4.16–26.16	4.02	16.79	7.50
		Overall	**11.25 ± 8.03**	**3.14**–**26.16**	**5.51**	**16.99**	**9.27**
	ASSD (mm)	RO_1_	3.19 ± 1.61	1.16–6.45	2.05	4.34	3.27
		RO_2_	3.10 ± 1.98	1.63–6.56	1.68	4.51	2.58
		Overall	**3.15 ± 1.76**	**1.16**–**6.56**	**1.89**	**4.40**	**2.75**

*Note*. The CI of the mean assumes sample means follow a *t*‐distribution with *N* – 1 = 9 degrees‐of‐freedom.

**FIGURE 7:**
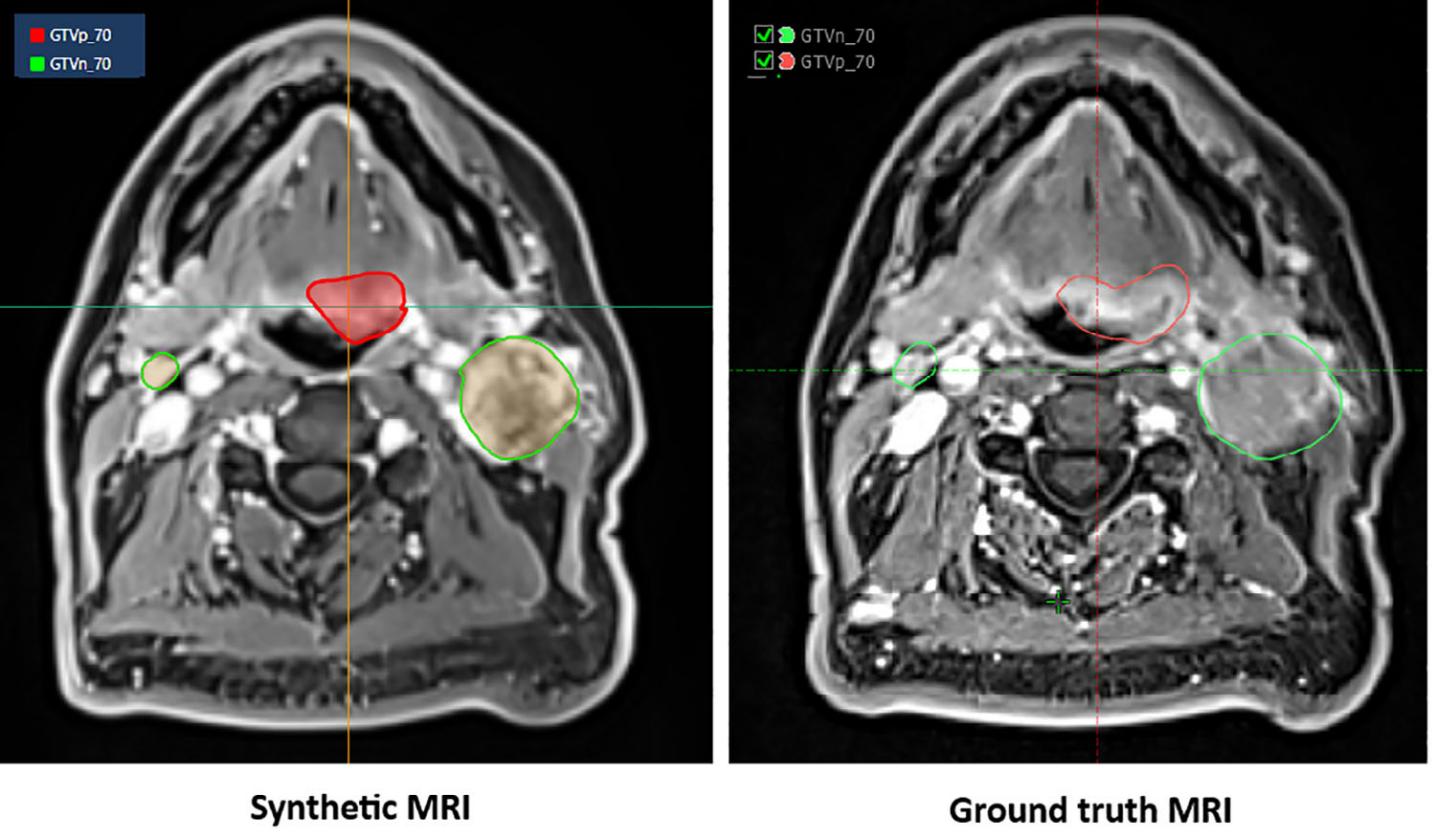
Representative comparison of GTVp_70 and GTVn_70 contours on matched axial anatomical location/image slice of sMRI and gtMRI. (a) pCT‐derived sMRI using the full Pix2PixGAN–diffusion pipeline; (b) Corresponding gtMRI. The GTVp_70 contour is outlined in red, and the GTVn_70 is shown in green.

## 4 DISCUSSION

This proof‐of‐concept study demonstrates that a two‐stage Pix2PixGAN–diffusion framework can generate sMRI in close agreement with the gtMRI in patients with H&N cancer. The CT images were acquired on two in‐house scanners (Philips^®^ Brilliance^™^ Big Bore, and Toshiba^®^ Aquilion^™^ Large Bore) with matched acquisition parameters (120 kVp, 2 mm slice thickness, 1.367 × 1.367 mm native in‐plane pixel spacing). The deliberate inclusion of multi‐vendor scanners was intended to enhance model robustness and generalizability within our departmental workflow. As CT is a quantitative imaging modality with standardized HU calibration, inter‐vendor intensity differences are expected to be minimal when acquisition parameters are matched. This dual‐scanner training strategy reflects realistic clinical deployment conditions at our institution, representing a strength of the proof‐of‐concept design rather than a confound requiring sub‐group correction. The reference gtMRI was a gadolinium‐enhanced T1‐weighted Dixon sequence. The goal of the synthesis framework was to investigate whether MRI‐like soft‐tissue contrast and enhancement‐related appearances could be synthesized directly from pCT, without requiring ceCT as an input. Although pCT does not directly encode contrast‐enhancement physiology, the model may learn statistical associations between CT‐visible anatomy and the corresponding appearance on gadolinium‐enhanced gtMRI. Therefore, the generated sMRI should not be interpreted as an exact or physiologically accurate representation of patient‐specific enhancement.

A key methodological concern in studies that rely on CT–MRI DIR for paired data generation is the quality of that registration. Landmark‐based TRE analysis across all cases yielded a mean TRE of 2.15 ± 1.19 mm (median 1.95 mm), consistent with literature values of 1 – 3 mm for H&N CT–MRI registration.^37^ Larger errors were observed at the basion and C3 vertebra landmarks in cases with greater patient motion, dental artifacts, or susceptibility‐related MR distortions, consistent with previously reported challenges in H&N CT–MRI registration.^7^, ^8^ The CT–MRI DIR was used to create the paired training dataset and, for this study, to bring the sMRI and corresponding gtMRI into the common spatial framework for voxel‐wise evaluation. In clinical deployment, however, the model requires only a single pCT image‐set as input. Any resampling to the model's expected input resolution is performed automatically as part of the standard pre‐processing workflow, without registration to a separate reference image, as no gtMRI will be available at the time of use.

The use of a diffusion‐based refinement stage to suppress GAN hallucinations is consistent with emerging literature.^19^ Early stopping in the reverse diffusion process, implemented here *via t* = 100/1000 initialization, has been shown to reduce hallucinations in medical image reconstruction by constraining the generative search space to a neighborhood around the conditional model estimate.^38^ This principle underlies recently published frameworks such as DynamicDPS,^39^ which demonstrated that initializing diffusion refinement from a conditional model prediction rather than pure Gaussian noise effectively reduces hallucinations while minimizing computational overhead. Furthermore, GAN‐based cross‐modality synthesis has been specifically shown to produce larger reconstruction residuals at tissue boundaries,^40^ the precise regions where our learned residual maps demonstrate the greatest Stage‐2 corrections, supporting the complementary role of the diffusion refiner in addressing known GAN limitations in H&N synthesis.^41^

The model produced a median MS‐SSIM of approximately 0.83 (mean 0.82 ± 0.02) and a median SSIM of approximately 0.71 (mean 0.70 ± 0.03), indicating that the sMRI captures a substantial amount of structural detail and broad soft‐tissue contrast present in the gtMRI. However, some differences are expected in low‐contrast areas and in slices affected by artifacts. Current literature is heavily focused on MRI‐to‐CT (sCT) synthesis, and, to date, there are no emerging data on CT‐to‐MRI synthesis for H&N cancers specifically. A study by Li *et al.* ^42^ employed a conditional GAN to generate sMRI from brain CT scans, yielding an SSIM of 0.94 and PSNR values of nearly 32 dB. Given that the brain's structure is far more homogeneous than that of the H&N, and that diagnostic MRI typically benefits from higher SNR and less geometric distortion, their better performance is expected. In contrast, our study focuses on H&N radiotherapy planning datasets, which pose challenges due to positional variation, dental artifacts, greater anatomical heterogeneity, susceptibility‐related distortions, geometric inconsistencies, and disease extent that varies between patients. A lumbar‐spine CT‐to‐MRI cycleGAN model achieved mean SSIM values as low as 0.28 and PSNR around 12 dB on its validation set,^43^ highlighting the difficulty of obtaining MRI‐like contrast when both structure and intensity change substantially. Douglass *et al.* ^44^ utilized Pix2Pix to generate cranial sMRI from optical surface images, achieving an MS‐SSIM of 0.83. Their input method differs substantially from CT; yet their results indicate that MS‐SSIM values above ∼0.8 demonstrate strong cross‐modality performance in challenging anatomical contexts. More broadly, cross‐modality medical image translation studies typically report SSIM values between 0.75 and 0.90 and PSNR values between 18 and 30 dB, though these values are highly dependent on anatomy, acquisition quality, and model architecture.^45^ Our results fall well within this published range. We also observed a relatively small difference between the mean and median scores for all four parameters: MS‐SSIM, SSIM, PSNR, and MAE, suggesting consistent model behavior across most slices. However, some outliers with low MS‐SSIM and SSIM values were observed, primarily in inferior neck slices and the shoulder region, as well as in slices affected by swallowing motion or dental artifacts. An MAE of ∼4 – 5% of the normalized intensity scale indicates that the generated sMRI replicates MRI signal intensities with reasonable fidelity. While our model was trained exclusively using pCT images, we also tested its performance on both pCT and ceCT image‐sets. For ceCT‐derived sMRI, PSNR decreased marginally, while MAE increased slightly. A closer examination of Figure 6 reveals that pCT‐derived sMRI has the potential to effectively highlight nodal volumes.

From a clinical perspective, it is encouraging that high SSIM and MS‐SSIM values were consistently observed in the oropharyngeal region, where GTVs and OARs are routinely contoured. Figure 3 shows that sMRI reproduces some of the key anatomical structures, such as mucosal surfaces, parotid glands, and the spinal cord, more clearly than CT alone, demonstrating promise for potential clinical applications. First, sMRI could assist centers with limited access to MR simulation by providing pseudo‐MR contrasts from pCT scans. Second, it may reduce the need for separate diagnostic or planning MR appointments. Third, sMRI could be integrated into the auto‐segmentation workflow to enhance the delineation of GTVs, with appropriate RO oversight.^46^

An exploratory scanner‐vendor comparison showed a small observed difference in MS‐SSIM (Philips^®^ 0.82 ± 0.06 vs. Toshiba^®^ 0.82 ± 0.07; difference 0.002), as detailed in the Results. However, given the small per‐vendor sample sizes, this should not be taken as evidence of multi‐vendor generalizability, which requires formal evaluation in the planned multi‐center validation phase.

The contour‐based analysis provides an initial clinical validation of the sMRI for GTV delineation. The GTVp_70 DSC (0.56 and 0.60) across the two evaluating ROs is lower than that of GTVn_70 (0.72 and 0.74), consistent with the inherently more challenging nature of primary tumor contouring due to irregular margins and mucosal involvement. The GTVn_70 DSC is broadly comparable to published inter‐observer variability data for H&N GTV delineation on MRI, where median DSC values of 0.73 – 0.76 for nodal volumes have been reported across experienced multi‐observer studies.^47^, ^48^ These studies consistently note that nodal volumes exhibit better inter‐observer agreement than primary tumors owing to clearer capsular boundaries. Our reported GTVp_70 DSC aligns with primary GTV segmentation benchmarks on MRI.^47^ The observed under‐segmentation tendency for GTVp_70 may reflect limitations in sMRI soft tissue contrast at the primary tumor margin. These results represent an encouraging preliminary step; further evaluation with experienced ROs and a larger cohort is warranted. This study did not include a CT‐only contouring arm, as CT‐ and MRI‐based GTV delineation are already known to differ substantially, with consensus guidelines recommending MRI for GTVs^49^, ^50^; the relevant question tested here is whether sMRI approximates true MRI‐based delineation when MRI is unavailable.

Despite these encouraging findings, we acknowledge that our study has a few limitations. This initial exploratory proof‐of‐concept study was restricted to 70 patients from a single institution. A further limitation is that the training and evaluation pCT images had been deformably registered with gtMRI to build matched pairs, so performance on native, unwarped pCT at deployment was not directly tested. The small registration error (mean TRE 2.15 ± 1.19 mm) indicates acceptable alignment, but validation on native CT remains an important next step. Our present study was limited to 2D slices, primarily to mimic the radiotherapy workflow, in which these slices are used to reconstruct 3D volumes in the planning system. While image‐level metrics such as SSIM, MS‐SSIM, PSNR, and MAE provide valuable global assessments, further clinical evaluation incorporating comprehensive GTV and OAR contour assessment, dose recalculation studies, and reader‐based image quality scoring would provide additional insight into the clinical utility of the proposed approach.

As this is a proof‐of‐concept study, thorough validation through multi‐center trials, independent clinical evaluation, and reader‐based assessment is required before this framework can be considered for routine clinical implementation. A prospective, blinded comparison of CT‐only vs. sMRI‐assisted delineation is identified as an important direction for future work. Future work will evaluate uncertainty estimation frameworks to propagate registration uncertainty into the synthesis quality assessment. Future studies will additionally focus on out‐of‐distribution detection and localized failure analysis across anatomical subregions, together with reader‐based perceptual scoring, to further establish the clinical robustness of the proposed framework. Further optimization of this model through multi‐institutional and multi‐vendor training and testing, followed by independent validation, would add substantial value to the emerging data in this field. The clinical utility of an sMRI solution derived from CT imaging would be far‐reaching, alleviating resource constraints in many clinical settings and enhancing the patient experience.

## 5 CONCLUSION

Our proof‐of‐concept deep‐learning model demonstrates the feasibility of generating sMRI from conventional radiotherapy pCT images, with close structural similarity to gtMRI. Landmark‐based TRE analysis indicated millimeter‐level spatial accuracy for the CT–MRI DIR used to align the image‐pairs. The proposed approach has the potential to supplement CT by assisting with delineation of GTVs for radiotherapy treatment planning and may be particularly valuable for centers that rely solely on CT‐based workflows. To build on this, further training and validation of the model on larger, multi‐center cohorts spanning a wider range of H&N subsites, tumor stages, and scanner protocols are warranted to improve robustness and generalizability before clinical adoption.

## AUTHOR CONTRIBUTIONS

**Prabhakar Ramachandran**: conceptualization, data curation, methodology, formal analysis, investigation, writing—original draft, writing—review & editing. **Akshay C. Dhargave**: formal analysis, writing—review & editing. **Catherine Jones**: data curation, investigation, writing—review & editing. **Dionee Lehman**: formal analysis, investigation, writing—review & editing. **Lachlan McDowell**: investigation, writing—review & editing. **Howard Liu**: conceptualization, methodology, investigation, writing—review & editing.

## CONFLICT OF INTEREST STATEMENT

The authors declare no conflicts of interest.

## FUNDING INFORMATION

The authors have nothing to report.

## ETHICAL APPROVAL

This retrospective study was approved by the Human Research Ethics Committee, Princess Alexandra Hospital (Metro South Health, Queensland Health), Woolloongabba, Queensland 4102, Australia (Approval No: HREC/2024/QMS/110045). All procedures were conducted in accordance with the Declaration of Helsinki and local institutional guidelines.

## Data Availability

The data supporting the findings of this study are not publicly available due to privacy and ethical restrictions but may be available from the corresponding author upon reasonable request and subject to institutional approval.
